# Optimization of the calculation of dietary energy intake in oncology chemotherapy patients: based on a longitudinal study

**DOI:** 10.3389/fnut.2026.1741202

**Published:** 2026-03-05

**Authors:** Yao Liu, Tian Zhang, Yufang Ren, Guorong Wang, Yanfen Wang

**Affiliations:** 1 Department of Neurology, Deyang People’s Hospital, Deyang, China; 2 School of Nursing, Chengdu University of Traditional Chinese Medicine, Chengdu, China; 3 Department of Nursing, Sichuan Clinical Research Center for Cancer, Sichuan Cancer Hospital & Institute, Sichuan Cancer Center, University of Electronic Science and Technology of China, Chengdu, China; 4 Department of Cardiovascular Surgery, West China Hospital of Sichuan University, Chengdu, China; 5 Department of Nursing, West China Fourth Hospital of Sichuan University, Chengdu, China

**Keywords:** chemotherapy, energy calculation, nutrition, oncology, optimization model

## Abstract

**Objective:**

The aim of this study was to investigate the current status of dietary intake in patients undergoing chemotherapy for oncology and to determine the types of dietary intake in patients undergoing chemotherapy for oncology in order to construct and validate an optimization model for dietary energy calculation.

**Methods:**

Using the convenience sampling method, tumor chemotherapy patients who were treated at a certain tertiary first-class tumor specialized hospital from January 2022 to January 2023 and met the inclusion criteria were enrolled. The 24-h dietary recall method was employed to conduct a longitudinal assessment of the patients’ dietary intake at five time points: before chemotherapy (T0), on days 1–3 of chemotherapy (T1), during the first week of chemotherapy (T2), during the second week of chemotherapy (T3), and during the third week of chemotherapy (T4). Hierarchical cluster analysis was used to classify the foods consumed by the patients undergoing chemotherapy. A multivariate linear regression analysis method was employed to establish an optimized model for calculating the dietary energy intake of patients with tumor chemotherapy. The goodness of fit and mean absolute error were used to evaluate the model.

**Results:**

A total of 116 patients were finally included in this study, and the dietary survey was completed for at least 4 cycles. Cluster analysis was performed based on the data of three characteristics of protein, fat and carbohydrate, and the results of the three-classification results were used to construct an energy calculation model for patients undergoing chemotherapy for tumors. R^2^ = 0.39, MAE = 265.83, the degree of fit and accuracy of the model is fair, suitable for clinical promotion and application.

**Conclusion:**

The energy calculation model in this study fitted reasonably well, and the model was evaluated well. The energy calculation model is simple to use and the food categories are easily accessible. The energy calculation model provides a convenient and efficient energy assessment tool for clinical nutritional management, which is conducive to promoting the dynamic monitoring of clinical patients’ dietary intake.

## 1 Introduction

Malignant tumors have become a significant health issue and a serious public concern that threatens human well-being and hinders societal development. They are the second leading cause of death globally, with the number of cases and fatalities continuing to rise each year (1). Studies indicate that 40%–80% of tumor patients suffer from malnutrition, and approximately 20% of patients die directly as a result of malnutrition (2, 3). The occurrence of malnutrition in tumor patients is mainly caused by the unique energy metabolism characteristics of tumor cells, chemotherapy-induced adverse effects, and digestive and absorption dysfunction (4, 5). Chemotherapy is one of the main therapeutic modalities for malignant tumors. While it can improve malnutrition through its anti-tumor effects, it may also cause or exacerbate malnutrition due to its adverse reactions. Chemotherapeutic drugs can cause nausea, vomiting, diarrhea, stomatitis, taste changes, gastrointestinal mucosal damage, loss of appetite, and anorexia. These effects indirectly reduce nutrient intake and absorption, further aggravating malnutrition beyond tumor-induced metabolic abnormalities (5). Thus, it is crucial to monitor the nutritional status of cancer patients undergoing chemotherapy. Conducting dietary survey is an important part of clinical nutritional management and assessment. A dynamic and quantitative assessment of the quantity and quality of nutritional intake is essential for the effective management of tumor chemotherapy patients.

However, existing challenges in clinical nutrition management complicate dietary and energy assessment. For example, the amount of precise clinical nutritional assessment is large, and nutritional assessment requires high professional knowledge of healthcare personnel (6), and the traditional energy calculation method is complicated, which reduces the efficiency of nurses’ work and patients cannot know whether their dietary intake is sufficient in time. In addition, during chemotherapy intervals, patients are discharged home to recuperate. Once side effects subside, they often resume a normal lifestyle but lack nutritional awareness (7). This leads to delayed nutritional assessment and support, increasing their nutritional risk. Healthcare professionals often pay insufficient attention to nutrition and have an inadequate knowledge base, which hinders clinical nutrition-related work (8). Therefore, there is an urgent need to develop a simple, fast, and operable energy calculation method for nutritional assessment.

Current methods of dietary survey and assessment include traditional approaches (e.g., dietary records, 24-h recalls, and food frequency questionnaires) and auxiliary tools (e.g., food maps, web-based surveys, mobile apps, and instant image analysis). The former involves complex calculations and demands high levels of expertise from researchers, creating inconvenience in clinical practice. The latter is limited by high technical requirements for image analysis, costly equipment, and narrow applicability, resulting in limited clinical adoption. Both traditional dietary surveys and energy calculation methods using dietary survey aids rely on food nutrient composition calculations, with no further optimization or improvement in their computational approaches.

Therefore, the aim of this study is to construct a dietary energy calculation optimization model for tumor chemotherapy patients, which can simplify the process of dietary energy, improve the compliance of patients with clinical dietary survey and assessment, and facilitate patients to understand their own eating situation at any time, so as to reduce the incidence of malnutrition risk in tumor patients, as well as simplify the process of calculating to improve the efficiency of clinical work.

## 2 Materials and methods

### 2.1 Study participants

A longitudinal study was conducted from January 2022 to January 2023 at Sichuan Cancer Hospital, the largest tertiary oncology hospital in southwest China. The study adhered to the Declaration of Helsinki and was approved by the Ethics Review Committee (No. SCCHEC-02-2022-098). Written informed consent was obtained from all participants or their family members.

Patients were recruited using convenience sampling. Inclusion criteria were: (1) hospitalized chemotherapy patients with pathologically confirmed malignant tumors and an expected survival ≥12 months; (2) age 18–70 years; (3) Karnofsky Performance Status (KPS) ≥ 70; and (4) voluntary participation. Exclusion criteria included inability to eat orally due to dysphagia, indwelling gastric tube, or other conditions, presence of distant metastases, or severe cognitive/speech impairment.

### 2.2 Dietary intake assessment

This study employed the 24-h dietary recall method, record the patient’s food intake (type of food eaten, name of food, composition, units, quantity, estimated amount, time and place of eating) for 3 consecutive days. The record included any food eaten other than the main meal, such as fruit, milk, nuts, and snacks. Quantitative accounting of patients’ dietary intake was performed using food weighing and food modeling, and daily energy intake (kcal/day) was calculated using MintNutrition dietary analysis software.

### 2.3 Data collection

The general information questionnaire was developed in consultation with clinicians, nutritionists, and statisticians, based on literature review and study objectives. The demographic information part includes: age, gender, ethnicity, occupational status, marital status, education level, monthly household income, health insurance payment method, history of smoking and alcohol consumption, etc. Disease-related data questionnaire included: time of disease diagnosis, tumor TNM stage, surgical method, time of surgery, disease duration, chemotherapy regimen, past history, recurrence and metastasis.

Prior to data collection, researchers received unified training from professional dietitians, including portion estimation, the use of food models, and the operation of food scales. The general information questionnaire was filled out by patients and their families and answering the questions of patients and their families when filling out the questionnaire in a timely manner. Completed questionnaires were reviewed on-site for accuracy and completeness. Clinical disease-related information was obtained through the electronic medical record system and checked with the patients. 24-h dietary questionnaires were filled out by the patients and their families according to the patients’ actual daily food intake, and were verified face-to-face by the researcher upon receipt of the patients’ completed dietary questionnaires. On the day of baseline data collection, the dietary intake of the patients on the 3 days before chemotherapy was investigated and filled in by the investigator. In addition, food scales were provided to patients free of charge to ensure the accuracy of dietary intake recording. The dietary intake of the patients at other time points was filled in by the patients themselves or their families in the 24-h dietary questionnaire. Patients who did not wish to fill in the form were asked the reason and recorded.

### 2.4 Sample size

The sample size was determined under repeated measurement conditions. The formula for sample size calculation is as follows: $n=\frac{{\left(Z_{\alpha \,/\,2}\right)}^{2}\,\pi \,\left(1-\pi \right)}{E^{2}}$ . The smaller the value of the allowable error, the better the estimation accuracy. The minimum required sample size was calculated with the maximum allowable error, α = 0.05, π = 0.5 when π(1−π) was taken as the maximum value, E was taken as 10%, and *n* ≈ 96 cases. Considering a 20% anticipated dropout rate, the sample size for this study was therefore at least 115 cases.

### 2.5 Statistics analysis

The survey data were entered and data managed using double checking, and the data were statistically analyzed using SPSS 23.0 as well as Python 3.11.2. A difference of *P* < 0.05 was used to indicate statistical significance. SPSS 23.0 was used to statistically describe the patients’ socio-demographic data, disease-related data, and dietary energy, measurement data conforming to a normal distribution were described as mean ± standard deviation and counts were expressed as percentages. Group comparisons were performed using ANOVA, with the Bonferroni employed to correct for multiple testing. Python 3.11.2 was used for food classification and the construction of a dietary energy calculation model. Specifically, food classification was performed using hierarchical clustering with Euclidean distance as the distance metric, while the dietary energy calculation model was established via multiple linear regression analysis.

## 3 Results

### 3.1 Patient characteristics

A total of 121 patients with chemotherapy for tumors were included in this study, and five patients who had completed only one dietary survey by the end of the study were excluded, resulting in a total effective sample size of 116 patients. The age of the patients ranged from 29 to 70 years old, with a mean age of (53.79 ± 9.96) years, as shown in Table 1. The treatment modality was mainly chemotherapy alone, which accounted for 84.5% of the patients, while a minority received immunotherapy or targeted therapy. Chemotherapy cycles were administered every 21 days, with a total of 4–6 cycles completed.

**TABLE 1:** Results of the socio-demographic profile of the study population.

Variables	*n*	Percent (%)
Sex		
Male	45	38.8
Female	71	61.2
Age		
<40	12	10.3
40∼59	74	63.8
≥60	30	25.9
Marital status		
Divorced	5	4.3
Married	106	91.4
Single	5	4.3
Education level		
Primary school	43	37.1
Junior high school	38	32.8
High schools and junior colleges	21	18.1
College and above	14	12.1
Smoking history		
No	87	75.0
Yes	29	25.0
Drinking history		
No	98	84.5
Yes	18	15.5
Tumor type		
Lymphomas	21	18.1
Gastric colorectal tumors	27	23.3
Tumors of the uterus and ovaries	40	34.5
Others	28	24.1
Disease stages		
I	15	12.9
II	43	37.1
III	53	45.7
IV	5	4.3
Type of treatment		
Radiotherapy	98	84.5
Targeted, immunotherapy + chemotherapy	18	15.5
Surgical treatment		
No	53	45.7
Yes	63	54.3
Fundamental disease		
No	17	14.7
Yes	99	85.3

### 3.2 Energy intake status

The results of the study showed that the energy intake of patients with tumor chemotherapy showed an overall trend of decreasing and then increasing with the cycle of chemotherapy. Patients consumed the least amount of energy on days 1–3 of chemotherapy (T1), and energy intake increased during the first week of chemotherapy (T2) and the second week of chemotherapy (T3), but energy intake was still lower than that before chemotherapy (T0). There was a significant difference in overall energy intake within each chemotherapy cycle (*P* < 0.001), after Bonferroni correction for multiple testing, the differences remained statistically significant (adjusted *P* < 0.05), as shown in Table 2.

**TABLE 2:** Energy intake of patients during chemotherapy (kcal).

Chemotherapy cycle	Time	*n*	DEI (kcal/day)		$\bar{x}\pm s$	F	*P*
			Min	Max			
1	T0	116	1039.19	1681.29	1332.01 ± 149.67	52.217	<0.001
	T1	116	731.27	1442.16	1071.78 ± 124.91		
	T2	116	884.6	1542.43	1194.86 ± 139.42		
	T3	116	913.89	1634.3	1250.71 ± 151.11		
	T4	116	941.68	1665.10	1266.35 ± 145.59		
2	T1	112	832.61	1350.60	1059.38 ± 113.14	40.792	<0.001
	T2	112	808.5	1534.86	1200.48 ± 148.32		
	T3	112	913.87	1554.46	1241.21 ± 140.74		
	T4	112	937.14	1692.23	1235.77 ± 128.65		
3	T1	111	787.21	1284.71	1068.63 ± 102.82	36.041	<0.001
	T2	111	915.48	1624.61	1185.43 ± 140.33		
	T3	111	905.31	1652.18	1230.23 ± 134.98		
	T4	111	957.75	1499.92	1236.20 ± 119.31		
4	T1	109	817.54	1437.81	1065.57 ± 112.37	39.552	<0.001
	T2	109	865.05	1456.09	1147.00 ± 121.81		
	T3	109	935.34	1448.61	1202.56 ± 114.82		
	T4	109	981.25	1610.92	1227.73 ± 124.21		
5	T1	61	806.40	1294.35	1062.54 ± 86.01	27.807	<0.001
	T2	61	910.82	1465.42	1155.05 ± 123.99		
	T3	61	991.57	1492.62	1211.88 ± 104.84		
	T4	61	1029.73	1550.56	1243.08 ± 123.91		

### 3.3 Establishment of food banks

The data came from the results of the longitudinal study dietary survey, and a food bank for chemotherapy patients with tumors was created in Excel, named “basic menu.” The energy and nutrients contained in 100 g of each food were recorded according to the food composition table, which mainly included 22 nutrients, including protein, fat, carbohydrates, dietary fiber, vitamins A, B1, B2, C, E, carotenoids, niacin, cholesterol, magnesium, calcium, iron, zinc, copper, manganese, potassium, phosphorus, sodium, and selenium. The Food Bank contains a total of 736 food items, including staple foods, meat and vegetarian dishes, soups, eggs and milk, fruits, nuts, and oral nutritional preparations.

### 3.4 Multiple linear regression analysis

**FIGURE 1:**
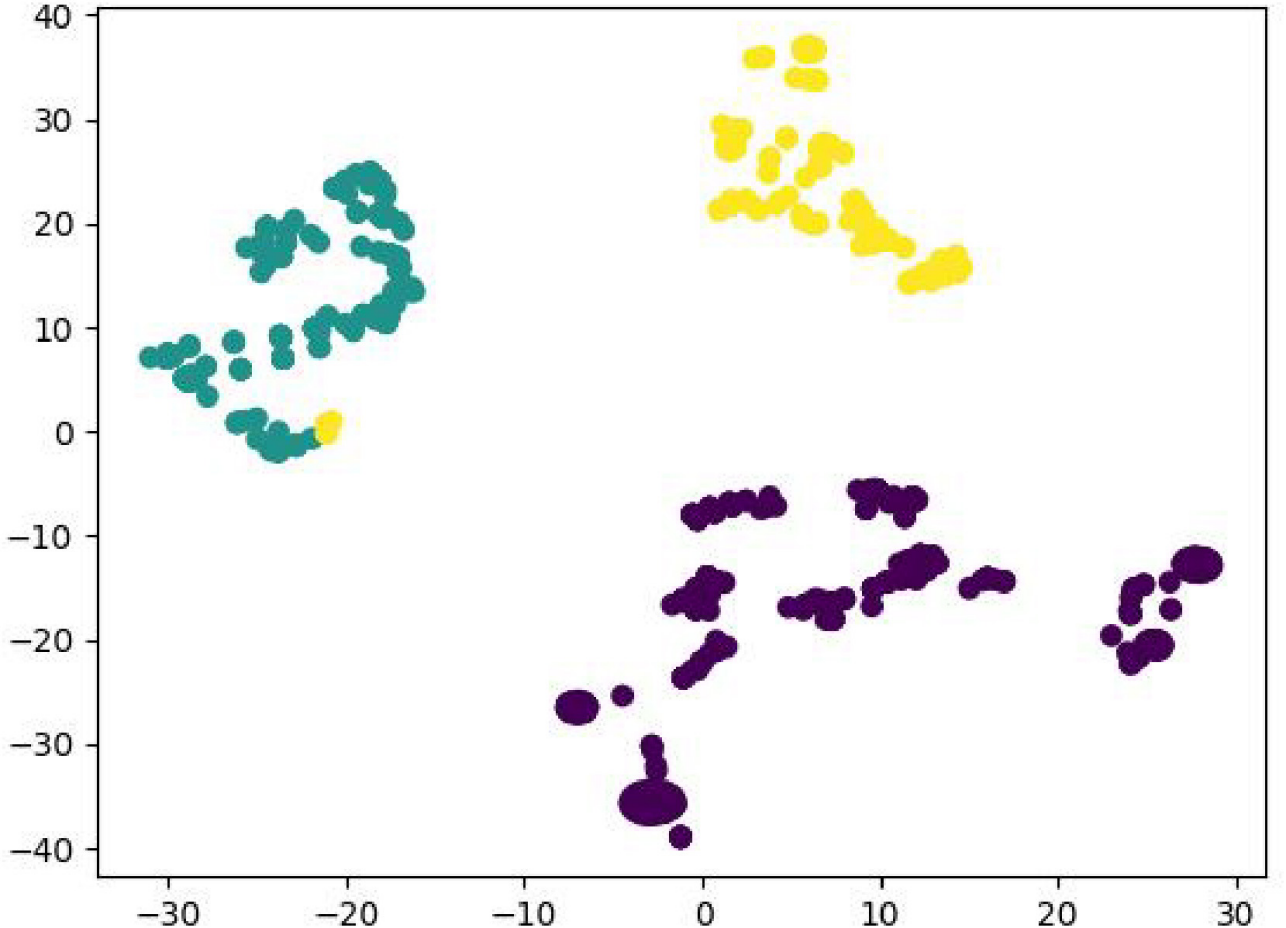
Schematic diagram of the triple classification.

**FIGURE 2:**
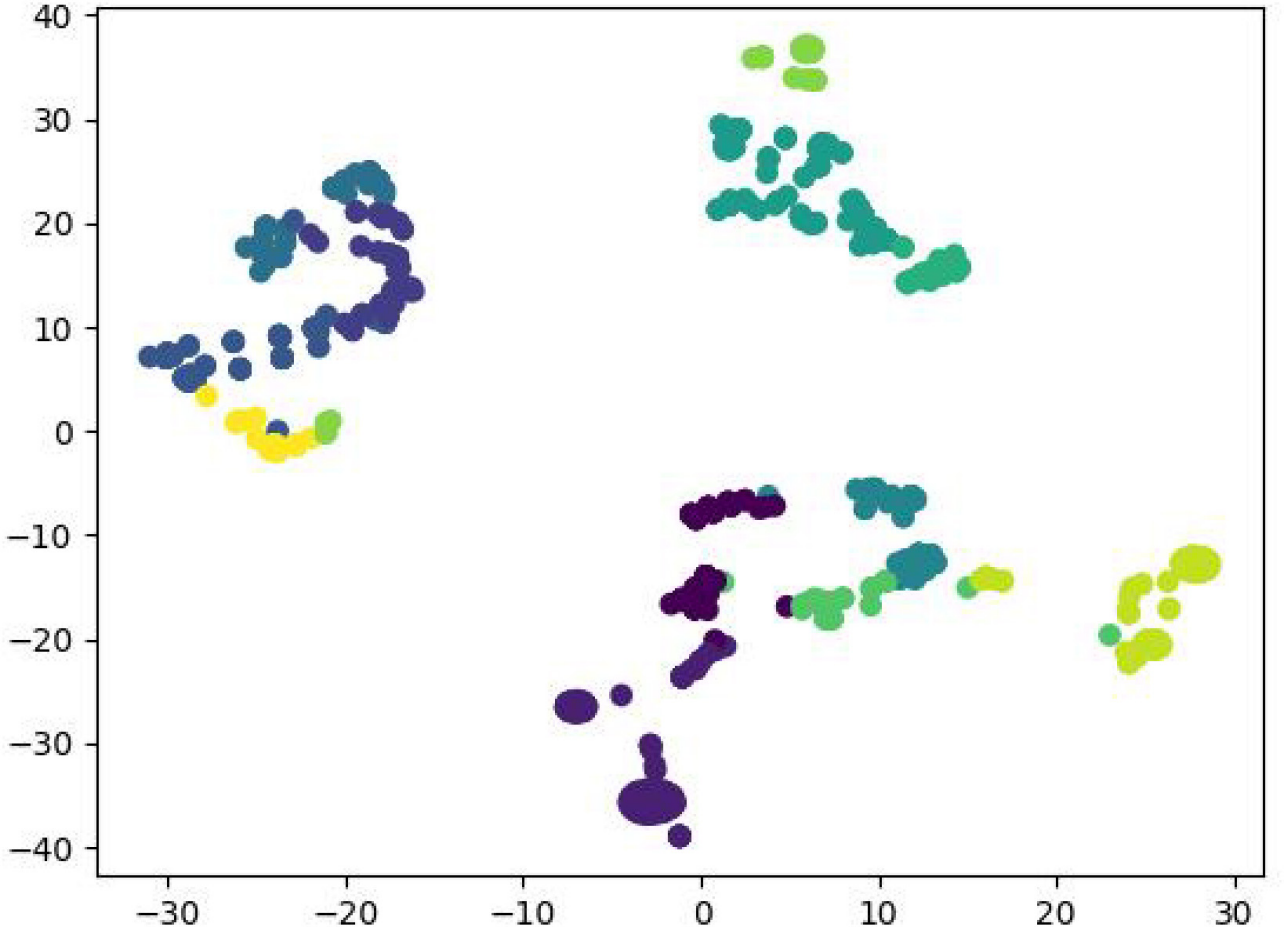
Schematic diagram of the twelve classifications.

Z-score normalization was applied to standardize food-related data in the food database, transforming the data into a standard normal distribution (mean = 0, standard deviation = 1), which allowed the variables to be compared under the same criteria, and at the same time reduced the influence of differences in magnitude and order of magnitude on the judgment of the distance between points. K-means was used to cluster analyze and classify the three characteristic attributes of protein, fat, and carbohydrate into nine classes, with the characteristic values replaced with 0–8. The Agglomerative Clustering function was used to perform a hierarchical cluster analysis of the foods and output the classification results. We finally compared the three classified and twelve classified food classification results (Figures 1, 2). A total of 2082 training data were generated by reading the data and predicting the categories and generating training data based on patient dish information. Linear Regression was used to build a multiple linear regression prediction model. The train_test_split function was used to randomly divide the dataset set into training set and test set according to the ratio of 7:3, and the final training set had a total of 1457 pieces of data and the test set had a total of 625 pieces of data. The coefficients corresponding to each food group were calculated using the Ir.coef function, and the intercept was calculated using the Ir.intercept function. The mean absolute error (MAE) was calculated using the metrics.mean_absolute_error function. The accuracy and effectiveness of the algorithmic models were measured and tested using the goodness-of-fit (*R*^2^) and mean absolute error (MAE), as shown in Table 3.

**TABLE 3:** Indicators for model evaluation.

Classification	*R* ^2^	*MAE*
Triple classification	0.39	265.83
Twelve classifications	0.55	231.45

The results of fitting the multivariate linear regression model based on the triple classification of food were as follows:


$$y=662.26+0.83\,x_{a}+1.02\,x_{b}+0.64\,x_{c}$$

The results of fitting the multivariate linear regression model based on the twelve classifications of food were as follows:


$$\begin{array}{c} y=335.66+1.16\,x_{a}+0.89\,x_{b}+2.16\,x_{c}+1.04\,x_{\text{d}}+0.98\,x_{e}+\\ 0.82\,x_{f}+1.18\,x_{g}+1.05\,x_{h}\\ +1.84\,x_{i}+0.60\,x_{j}+0.49\,x_{k}+0.69\,x_{l} \end{array}$$

*y* represents energy (kcal), *x*_*a*_,*x*_*b*_…*x*_*l*_ represent the food weights (g) for food categories a, b, …, l.

## 4 Discussion

The results of the study showed that patients had the lowest dietary intake on days 1–3 (T1) of chemotherapy during a cycle of chemotherapy, as time progressed after chemotherapy, dietary energy intake gradually increased and eventually returned to near pre-chemotherapy levels. The findings indicate that the early stage of chemotherapy is a critical period for nutritional intervention. Accordingly, nutritional education should be implemented early during chemotherapy to improve self-awareness of nutrition among patients undergoing cancer treatment, with the goal of ensuring that dietary energy intake meets the target or recommended intake and maintaining optimal nutritional status. According to the Practice Guidelines for Nutritional Therapy of Oncology Patients issued by the European Society of Clinical Nutrition and Metabolism (ESPEN) (9), the recommended dietary intake of oncology patients undergoing chemotherapy is 25–30 kcal/d, and the target energy intake is calculated according to the body mass of the patients. Therefore, nursing staff should individualize the target amount and provide nutritional guidance when conducting nutritional assessment and intervention. Furthermore, this study found that patients’ dietary intake had largely recovered to pre-chemotherapy levels by the third week (T4) of chemotherapy. This may be attributed to the gradual clearance of chemotherapeutic agents from the body over time, resulting in reduced side effects, improved appetite, relief from nausea and vomiting, and consequently increased dietary intake.

This energy calculation model can optimize the personalized management and monitoring of insufficient dietary nutrient intake in oncology chemotherapy patients, simplify the clinical dietary assessment procedure, and improve the efficiency of healthcare work. Ensuring adequate dietary energy intake during chemotherapy is of great significance to oncology patients, and understanding the patient’s energy intake is the basis of nutritional intervention. At present, clinical dietary surveys mainly use the 24-h dietary review method, the dietary frequency method and the food exchange portion method, etc. The energy assessment mostly relies on the traditional accurate calculation of food nutrients, but this method requires high professionalism of researchers, has a large amount of workload, and the calculation process is cumbersome, which is not applicable to the monitoring of patients’ self-energy intake and the nurses’ rapid assessment of the patients’ energy intake. Some studies at home and abroad have used APP to record dietary intake, such as the Mint Nutrition APP, the application can automatically calculate energy based on the food name entered by the patient as well as the amount of food eaten, but the method requires patients to familiarize themselves with the use of electronic smart devices, which is not clinically universal, and the actual energy method based on the APP is the same as that of the traditional precise algorithm. Hanping Shi developed a concise dietary self-assessment tool for tumor patients, which quantifies the common dietary patterns in China and designs different versions of self-assessment tables according to the characteristics of regional staple foods, but the method dietary patterns have problems such as insufficient comprehensibility and the validity of the tool to be verified, which has not yet been widely used in the clinic (10). Therefore, there is a lack of simple and operable energy calculation methods and tools.

In this study, we categorized foods through cluster analysis and constructed an energy calculation model with food category as a variable, breaking the traditional energy calculation method that relies on the nutrient composition of foods, and providing a more convenient and operable energy assessment method for clinical health care personnel and patients, in order to promote patients’ attention to the nutritional status, and to advance the development of clinical nutritional management.

In this study, dietary intake data of tumor chemotherapy patients were collected longitudinally, including a total of 736 food items, 23 food component features, and about 60,000 pieces of dietary intake data. Considering energy as well as food nutrients, protein, fat, and carbohydrates were ultimately selected as feature attributes for food clustering analysis, and a food trichotomous classification-based energy computation model was constructed, which enhanced the reliability of the study.

After model fitting, the model fitting effect and accuracy were evaluated using the goodness-of-fit *R*^2^ and the mean absolute error MAE. the closer the *R*^2^ was to 1, the better the model fitting effect was, and the mean standard error MAE was used to evaluate the gap between the model predicted values and the actual observed values, and the smaller it was, the better the accuracy of the model was. The model based on food trichotomies in this study had *R*^2^ = 0.39 and MAE = 265.83, with good food clustering. The energy calculation model based on food trichotomies is suitable for clinical applications and nutritional assessment. There are several limitations of this study: (1) this was a single-center observational study using convenience sampling, which inevitably introduces selection bias and limits sample representativeness, potentially affecting the generalizability of the results. (2) Although food scales were provided to the patients in this study, some patients’ compliance was poor, and the quality of the dietary questionnaires was not high; the dietary surveys for some patients before 1 cycle of chemotherapy (T0) were filled out based on patients’ recollections, which may affect the accuracy of the results due to memory bias.

## 5 Conclusion

The early stages of chemotherapy are a crucial period for providing nutritional health education and intervention for patients undergoing tumor chemotherapy. During the first 3 days of chemotherapy, patients often experience the most significant decrease in dietary intake due to the effects of chemotherapeutic drugs. Therefore, healthcare professionals should implement early interventions that address these adverse effects while closely monitoring changes in patients’ dietary intake. In this study, we developed a dietary energy calculation model based on food categories. This model includes three food category parameters and food weights, along with a constant term. The energy calculation model is straightforward and easy to use. It has been trained on 1,457 data points and validated on 625 data points, demonstrating good predictive ability. This model can serve as a valuable reference for clinically assessing dietary energy intake and determining whether the intake of tumor chemotherapy patients meets established standards.

## Data Availability

The raw data supporting the conclusions of this article will be made available by the authors, without undue reservation.
